# Biomaterial-based strategies for maxillofacial tumour therapy and bone defect regeneration

**DOI:** 10.1038/s41368-021-00113-9

**Published:** 2021-03-16

**Authors:** Bowen Tan, Quan Tang, Yongjin Zhong, Yali Wei, Linfeng He, Yanting Wu, Jiabao Wu, Jinfeng Liao

**Affiliations:** State Key Laboratory of Oral Diseases & National Clinical Research Centre for Oral Diseases & West China Hospital of Stomatology, Sichuan University, Chengdu, China

**Keywords:** Oral cancer, Drug development

## Abstract

Issues caused by maxillofacial tumours involve not only dealing with tumours but also repairing jaw bone defects. In traditional tumour therapy, the systemic toxicity of chemotherapeutic drugs, invasive surgical resection, intractable tumour recurrence, and metastasis are major threats to the patients’ lives in the clinic. Fortunately, biomaterial-based intervention can improve the efficiency of tumour treatment and decrease the possibility of recurrence and metastasis, suggesting new promising antitumour therapies. In addition, maxillofacial bone tissue defects caused by tumours and their treatment can negatively affect the physiological and psychological health of patients, and investment in treatment can result in a multitude of burdens to society. Biomaterials are promising options because they have good biocompatibility and bioactive properties for stimulation of bone regeneration. More interestingly, an integrated material regimen that combines tumour therapy with bone repair is a promising treatment option. Herein, we summarized traditional and biomaterial-mediated maxillofacial tumour treatments and analysed biomaterials for bone defect repair. Furthermore, we proposed a promising and superior design of dual-functional biomaterials for simultaneous tumour therapy and bone regeneration to provide a new strategy for managing maxillofacial tumours and improve the quality of life of patients in the future.

## Introduction

Maxillofacial tumours are a general term for tumours occurring in the neck and face^1–3^ (including the oral cavity, salivary glands, and temporal-mandibular joint) and are classified into three main categories: odontogenic tumours, general tissue-derived tumours, and maxillofacial cysts. Histologically, malignant general tissue-derived tumours are mostly categorized as squamous cell carcinoma^4^ (~80%), which has highly destructive, aggressive and metastatic tendencies, and were responsible for 177 384 deaths in 2018 worldwide^5^. In addition, odontogenic tumours^6^ derived from the odontogenic epithelium and mesenchymal tissues, along with the maxillofacial cysts^7,8^ from soft tissue and jaw bone, are mainly benign tumours that are relatively less invasive and show slow growth. However, maxillofacial tumours and their treatment might be destructive, whether physically or psychologically, resulting in substantial tissue loss (including skin, adipose tissue, muscle, bone) and a reduction in quality of life. The world’s annual investment in maxillofacial management has been increasing. Nevertheless, the burden of a series of related treatments (e.g., postsurgical reconstruction^9^) owing to tumours is still increasingly heavy.

Traditional clinical treatments for maxillofacial tumours include surgery, radiotherapy, and chemotherapy. Unfortunately, current clinical therapies are destructive to hosts, resulting in unsatisfactory therapeutic outcomes as follows: (1) Physiological dysfunction^10–12^, including issues with chewing, speech, swallowing, sucking, and breathing, owing to the lack of maxillofacial bone and radiation and chemotherapeutic drug damage; (2) Psychological damage resulting from the impairment of normal physiological functions and maxillofacial deformity^13,14^, which could lead to depression and misanthropy in patients; and (3) Frequent tumour recurrence due to cancer cells that survive surgery, inaccessibility of tumours during operations, unpredictable variation and constantly increasing drug resistance. Some novel methods, such as immunotherapy^15,16^, have shown strong potential, particularly biomaterial-mediated therapies (including targeted chemotherapy, magnetic-mediated hyperthermia (MMT), photodynamic therapy (PDT), and photothermal therapy (PTT)), which have demonstrated high anticancer efficiency, specific tumour targeting properties, excellent biocompatibility and low invasiveness. Different scales of biomaterials have various anticancer mechanisms. Nanomaterials show favourable release kinetics and pharmacokinetics of drugs^17^, can control localized hyperthermia^18^ and radiation^19^, allow enhanced tumour permeation and retention^20^, and achieve precise tumour imaging and targeting^21^. Macromaterials can control drug release at a macroscopic level^22^; more crucially, macromaterials serving as cell-growing scaffolds facilitate the tissue repair process. These biomaterial-mediated techniques are promising options for tumour management because they eliminate primary cancer cells and prevent recurrence following tissue reconstruction.

A major issue is the bone loss issue caused by tumour invasion, which strongly affects the quality of life of patients. A constant equilibrium of bone resorption and formation is needed for the highly dynamic and complicated maxillofacial skeleton system^23^, and the bony structure is destroyed when exposed to disruptive factors. Aggressive maxillofacial malignant tumours often encroach on bone tissue by creating a bone-destructive immune environment^24^, and some benign tumours with slow growth properties also cause large bone-occupying lesions due to their large size, thereby exerting adverse effects on the maxillofacial morphology and function of patients. Autologous, allogeneic, and xenogeneic grafts, prostheses and tissue engineering are general bone repair techniques. In current clinical practice, autologous grafts are a relatively common and efficient method. In 1975, Taylor et al.^25^ proposed applying fibula flaps for long bone reconstruction, and in 1989, this technique was first reported for oromandibular rebuilding by Hidalgo et al.^26^, who described a quintessential autologous bone graft (fibula flap) worthy of being cited. Fibula flaps present as vascularized bone tissue removed from the fibula, with a suitable bone density and modulus, and more importantly, autologous materials do not cause immune rejection, preventing collapse of the materials due to the immune response and maintaining the stability of osseointegration. However, autograft bone also has some risks and challenges, such as the impairment of normal body functions, weakness and instability of the ankle, fibula and feet, and tibial stress fracture^27^. Accordingly, clinicians and researchers are still faced with the challenge of how to improve the outcome of autograft repair.

**Fig. 1:**
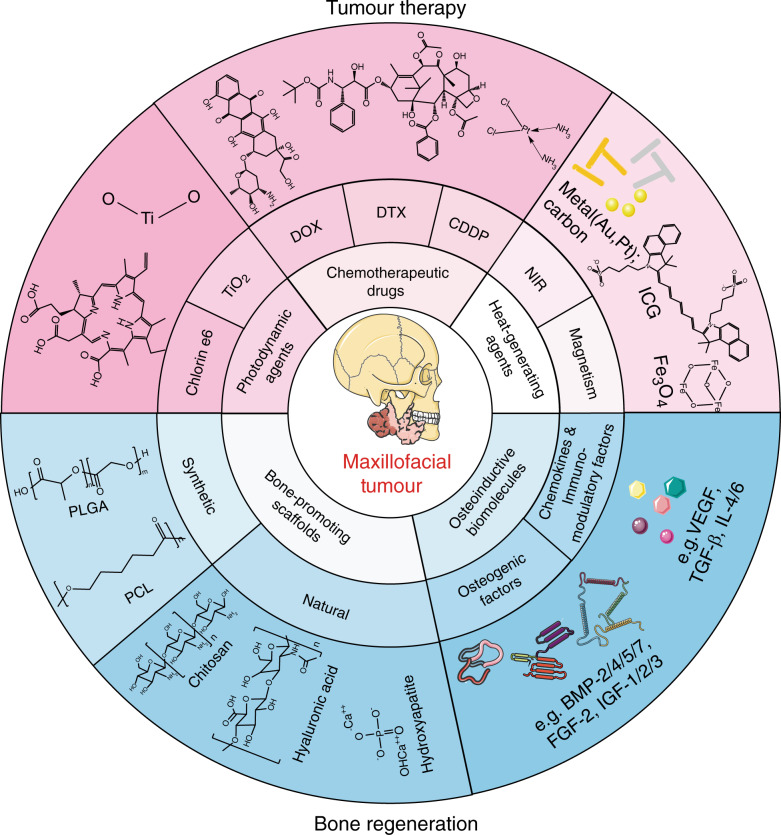
Bioactive material scaffolds designed for maxillofacial tumour therapy and bone regeneration. Photodynamic agents, chemotherapeutic drugs, and heat-generating agents are the three main parts of biomaterial-mediated tumour therapy (the upper part of the figure), while scaffold materials are also bone-promoting and osteoinductive biomolecules that stimulate the bone formation process (the lower part of the circle), indicating the potential of this scaffold in bone regeneration. DOX, doxorubicin; DTX, docetaxel; CDDP, cisplatin; ICG, indocyanine green; PLGA, poly(lactic-co-glycolic acid); PCL, polycaprolactone; NIR, near infrared ray; BMP, bone morphogenetic protein; FGF, fibroblast growth factor; IGF, insulin-like growth factor; IL-4, interleukin-4; TGF-β, transforming growth factor-β

In recent years, with the continuous development of biomaterials, the field of repairing maxillofacial bone defects has shown considerable progress. Generally, biomaterials for bone reconstruction are classified into bioinert and bioactive materials. Bioinert materials refer to materials with slow degradation rates, provisional support and similar mechanical properties to bone tissue, including obturator prostheses (e.g., rubber and silica gel) and biocompatible metals (e.g., titanium^28^ and magnesium). Obturator prostheses can be utilized to temporarily fill substantial maxillofacial bone defects and act as long-term fillers. However, the lifespan of these prostheses could be halved owing to disturbance from the in vivo microenvironment, which could increase the risk of infection and cause additional trauma resulting from prosthesis removal and reimplantation during secondary surgery. However, bioactive materials stimulate and facilitate bone tissue regeneration by providing supportive scaffolds (chitosan, calcium phosphate, hyaluronic acid, etc.) and osteoinductive stimulatory factors (growth factors, bone extract, etc.) for cells^29^(e.g., mesenchymal stem cells, MSCs) to adhere to, proliferate and differentiate, thus creating a microenvironment called a “bioreactor”. Compared with bioinert materials, these materials can results in newborn bone, and from the perspective of repair outcomes, it is almost restored to the original state and can normally perform its function. Ideally, we would like to introduce dual-functional or multifunctional bioactive materials for maxillofacial tumour therapies as well as the subsequent bone regeneration to address two issues at once. Herein, Fig. 1 shows bioactive scaffolds designed for tumour treatment and bone regeneration, along with some representative elements included in this system.

Herein, this review provides a brief introduction to traditional tumour treatment and bone repair. Then, biomaterial-mediated maxillofacial tumour therapy and strategies for bone regeneration are highlighted. We also analyse the current challenges of using biomaterials in maxillofacial bone repair. Finally, a possible treatment scheme and future clinical translation are proposed.

## Maxillofacial tumour therapy

At present, the treatment of maxillofacial tumours is generally based on surgery, and radiotherapy and chemotherapy are also clinically adopted. The therapeutic effect in benign tumours is satisfactory, but the outcome of malignant tumours is often discouraging. Currently, we are working on more effective and less invasive strategies, among which biomaterial-mediated therapy has shown potential. Herein, in Section 2.1, our review will cover traditional maxillofacial tumour therapies (mainly focused on malignant tumours). Then, biomaterial-mediated tumour therapy will be highlighted in Section 2.2.

### Traditional tumour therapy

The treatment of benign maxillofacial tumours (mostly odontogenic tumours and maxillofacial cysts) is primarily surgical^30^. Due to their slow growth and invasion, lesions and surrounding tissue are often removed. Although recurrence is possible, it can be well controlled. For malignant maxillofacial tumours, however, the survival rate is suboptimal. At present, surgery, chemotherapy, and radiotherapy are the three main treatment methods. In clinical practice, the design of an individualized therapeutic scheme needs to strictly follow the principles of the guidelines and consider the specific circumstances of the patient, including tumour-node-metastasis (TNM) staging^31^, according to some necessary auxiliary examinations (e.g., assessment of physical condition, biopsy^32^, imaging examination^33^). Surgery (radical resection) is regarded as the mainstay treatment. However, determining the edges of the carcinoma, avoiding damage to important blood vessels as well as nerves, and retaining as much normal tissue as possible in an attempt to prevent postoperative tumour recurrence and decrease surgical invasion^34,35^ are nonnegligible issues for surgical oncologists. For oral cancer in the early stage (I–II, without lymph node metastasis), surgery might be sufficient; nevertheless, for tumours at a more advanced stage (gross tumour volume, localized lymph node metastasis), surgery plus postoperative radiotherapy, which can eliminate residual tumour tissues due to incomplete resection or inaccessibility during the operation, would be preferred. By destroying the deoxyribonucleic acid (DNA) structure of cells in a wide range (cancer cells and normal cells)^36^, radiation therapy is effective in removing almost all tumour cells from the tumour and suspicious surrounding areas. Unfortunately, a series of complications, such as mucositis^37^, xerostomia^38^, and myelosuppression^36,39^, subsequently occur. Chemotherapy is more often recommended in patients diagnosed with stage III-IV disease, in which surgery and radiotherapy might not function efficiently and may only aggravate the situation. CDDP, paclitaxel (PTX), DTX and 5-fluorouracil (5-Fu) are representative chemotherapeutic drugs applied in the clinic. With chemotherapy, tumour progression and expansion are postponed, thus prolonging the patient’s lifespan. However, the toxicity to patients is a difficult issue to address and can also cause destruction of body systems. Chemotherapy and radiotherapy are both the standard postsurgical treatments to reduce the recurrence caused by residual malignant cells, but the eventual therapeutic results are still unsatisfactory.

Other adjuvant therapies, such as immunotherapy^40^, gene therapy and traditional Chinese medicine therapy^41,42^, can also be promising options. However, there are still many issues (mechanism, safety, effectiveness, ethics, etc.) that remain unsolved, and due to the stringent requirements of technical conditions, the spread of these methods has been severely limited. Broadly speaking, we believe that in the future, these technologies will be able to achieve accurate and personalized tumour treatment and substantially improve the survival rate and quality of life of cancer patients.

### Biomaterial-mediated tumour therapy

The limitations and disadvantages mentioned above, which might compromise the outcome of maxillofacial tumour therapy, indicate the need for novel methods and strategies. Luckily, the advent of biomaterial technology has made it possible to overcome the existing defects and provide a platform to integrate various treatment strategies. Biomaterials can be used as carriers to encapsulate drugs and improve their biodistribution and pharmacokinetic properties. By incorporating targeting elements such as monoclonal antibodies and receptor-specific peptides, targeted delivery can be achieved to improve the accumulation of drugs in tumour sites and improve the specificity and accuracy of treatment. Based on traditional therapies (e.g., chemotherapy, radiotherapy) as well as hyperthermia and reactive oxygen species (ROS) dynamic therapy, biomaterials can better control the treatment scope and intensity and effectively reduce adverse reactions to enhance the treatment efficiency. Moreover, with the participation of bioactive materials, the rate of tumour recurrence and metastasis may be effectively constrained. Targeted modification, novel drug delivery systems^43^ and specific treatment can maximize the tumour-killing effect and minimize dose-dependent adverse effects. Compromised by serious complications, conventional chemotherapy and radiotherapy are difficult to use in long-term treatments of patients, while biomaterial-mediated therapy can be a substitute for these methods, decreasing the incidence of recurrence.

### Biomaterial-mediated chemotherapy

Chemotherapy is relatively common, and biomaterials could result in an improvement in anticancer efficiency. Traditional chemotherapy has long been limited due to drug resistance and the severe side effects caused by the off-target toxicity of drugs. Fortunately, nanomaterials^44,45^ act as precise regulators for the control and release of chemotherapeutic drugs and not only maximize the accuracy of targeting tumour cells but also reduce the adverse reactions caused by drug leakage. DTX^46^ is a commonly applied anticancer drug that inhibits physiological microtubule depolymerization and disassembly, but its poor water solubility has restricted its application. After encapsulation of DTX into PLGA nanoparticles (NPs)^47^, improved drug delivery efficiency and reduced systemic side effects were observed. A release kinetics studies revealed a prolonged release period (60% of the total, at day 9), and the MTT test results of the SCC-9 human tongue carcinoma cell line also indicated enhanced cytotoxicity of DTX-loaded PLGA-NPs. Interestingly, in addition to PLGA, other components, including PVA and PEG, led to a negatively charged surface, which bypassed the disruption of the reticuloendothelial system, thus increasing the antitumour efficacy. Based on the fact that chitosan (CS)-based materials are promising carriers for drug delivery^48,49^, especially due to the improvement in water solubility, Ilaria Cacciotti et al.^50^ synthesized 18-β-glycyrrhetic acid (GA)-loaded CS-PLGA-based nanoparticles (GA-NPs) for OSCC treatment. By encapsulating GA into nanoparticles, the researchers could efficiently manipulate and locally concentrate the release. Interestingly, in the cytotoxicity test, GA demonstrated specificity for cancer cells (PE/CA-PJ15 cells) compared with normal human gingival fibroblasts (HGFs) in the control group, indicating a potential precisely targeted and efficient drug delivery system in future oral cancer treatment.

In addition to nanomaterials, materials at a more macroscopic scale show potential in improving antitumour routes and techniques. A microneedle technique^51^ was used to deliver lipid-coated CDDP nanoparticles (LCC-NPs) via the transdermal route to treat oral squamous cancer, and the apoptotic index (58.6%) of cancer cells, as well as the low systemic toxicity (including nephrotoxicity, pulmonary toxicity, and hepatotoxicity), confirmed the efficiency and safety of this anticancer treatment. In addition, as three-dimensional water-rich materials, hydrogels have an excellent drug loading capacity and biocompatibility and are ideal tools for improving anticancer drug control and release kinetics^52–54^. A self-assembling peptide hydrogel (ac-(RADA)_4_-CONH_2_)^55^ designed by Christina Karavasili et al. was applied for codelivering curcumin and DOX to treat head and neck cancers. With the participation of hydrogel, the release of curcumin and DOX was manipulated based on the aqueous solubility of these two drugs, and then, the enhanced synergistic anticancer effect on HSC-3 oral squamous cells was confirmed in vitro (the apoptotic cell number determined by flow cytometry was increased from 56% at 48 h to 73% at 72 h). The in vivo animal experiment results also indicated the strong anticancer effect, demonstrated by the shrinkage of the tumour volume compared with that of the control (saline group), and this hydrogel composite effectively reduced systemic cytotoxicity (20% body weight decrease in the curcumin/DOX solution delivery group, while the hydrogel composite group managed to maintain the body weight), which indicated the potential of reducing damage to the body system while ensuring the effect of tumour treatment.

#### Biomaterial-mediated photodynamic therapy

**Fig. 2:**
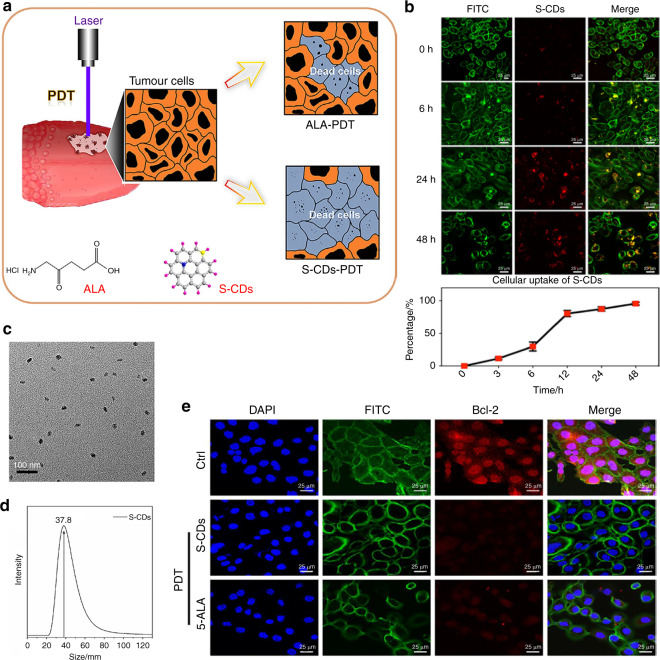
Sulphur-doped carbon dots for oral squamous cell carcinoma photodynamic therapy. **a** Illustration of the S-CDs and 5-ALA-mediated PDT in UM1 cells. **b** UM1 cellular uptake of S-CDs. UM1 cells were treated with S-CDs for 0, 6, 24, and 48h (1 µmol/L). Immunofluorescence images (S-CDs: red, cytoskeleton: green). Scale bars are 25 μm. Cellular uptake of S-CDs shown by flow cytometry. Data are presented as the mean ± SD (*n* = 3). **c** TEM image. **d** Dynamic light scattering. (**e**) After exposure to visible light and S-CDs and 5-ALA (40 nmol/L), immunofluorescence images of UM1 cells were obtained. ^58^Copyright 2020, Wiley

PDT^18,56^ relies on photosensitizers to generate ROS under irradiation with external light at a specific wavelength to kill tumours. Since maxillofacial tumours usually occur in areas with easy access to light irradiation, PDT can be conveniently implemented. With the involvement of targeted surface functionalization, photosensitizers can specifically accumulate at the tumour site. ROS-induced cancer cell apoptosis is the main mechanism of PDT, and for oral cancer treatment, this strategy can function well with the mediation of biomaterials. Toluidine blue O (TBO) is a photosensitizer with a high yield of singlet oxygen (^1^O_2_) generation, and Graciano et al.^57^ encapsulated TBO in a chitosan hydrogel (CS gel) to treat buccal cancer, which was effective. TBO retention was enhanced by CS gels (4 and 5% CS gels shared similar TBO release rates), thereby prolonging the valid period of PDT. After in vitro and in vivo studies, 4% CS gel was shown to have a relatively suitable pH for the mouth and rheological behaviour, indicating it is a promising tool that mediates PDT in oral cancer treatment. For a long period of time, researchers were plagued by the problems of inevitable phototoxicity and nonideal singlet oxygen (^1^O_2_) quantum yields. Li et al. designed novel sulphur-doped carbon dots (S-CDs)^58^, and the positive charge on the surface of the S-CDs facilitated UM1 cell uptake. Interestingly, to verify the PDT effect, researchers used a known and effective PDT agent, 5-aminolevulinic acid (ALA), for comparison. In ^1^O_2_ quantitative analysis, a high quantum yield of ^1^O_2_ of S-CDs (approximately 60.0 k, compared with 5.0 k in other non-S-CD groups) was observed, showing the abundant resources of ^1^O_2_. Particularly, the high PDT efficiency of S-CDs (strong fluorescent signals of Bax and caspase-3, which are Bcl-2 family proteins in the cell apoptosis pathway^59^) was attractive, indicating that S-CDs were a potential nanomaterial for PDT in the treatment of oral cancer (Fig. 2).

Moreover, two-photon active photosensitizers that release lethal ROS for nucleus-targeting PDT were also proven feasible in oral cancer therapy. Nasrin et al.^56^ fabricated novel conjugated carbon dots (CDs) combined with curcumin and folic acid to enhance internalization; accordingly, elevated cancer cell destruction was observed via a direct attack on DNA. In addition to carbon dots, iron oxide nanoparticles show potential for PDT; additionally, their imaging capacity is worth studying. Wang et al.^60^ designed a PDT platform consisting of tumour targeting ligand (Fmp), second-generation PDT drug (Pc 4), and iron oxide (IO) NPs. By encapsulating Pc 4 into the NPs, researchers found that a 10-fold lower dose of Pc 4 was less harmful yet still effective. Furthermore, the targeted Fmp-IO-Pc 4 NPs were more effective than nontargeted IO-Pc 4 NPs in restricting tumour growth, implying that the codelivery of Pc 4/Fmp by IO NPs can heighten the antitumour effect (head and neck squamous cell carcinoma (HNSCC) cells) while reducing the PDT drug dose to guarantee safety. Herein, in future clinical practice, targeted Fmp-IO-Pc 4 NPs might be a multifunctional therapeutic platform including PDT and magnetic resonance imaging (MRI).

In addition to light-induced ROS production, ultrasound can induce certain substances to produce ROS and free radicals. For instance, sonodynamic therapy (SDT) based on high-intensity focused ultrasound (HIFU) and TiO_2_ was designed to treat OSCC by S. Moosavi Nejad et al.^61^ Significant cell death was observed only in the HIFU group and the HIFU + TiO_2_ group, while the latter showed a greater destructive effect. HIFU + TiO_2_ resulted in a relatively stronger oral cancer cytotoxic effect in vitro, with a tendency to increase at higher TiO_2_ concentrations and HIFU intensities, and these results can be explained by the fact that HIFU promoted the cytotoxic effect of TiO_2_, which had a destabilizing effect on cell membranes. In vivo experiments showed that TiO_2_ particles accumulated in the tumour cytoplasm when exposed to HIFU, indicating that HIFU could induce TiO_2_ to penetrate the cell membrane. However, the team did not conduct experiments to evaluate the association between ROS production and enhanced cytotoxic effects, but previous studies have shown that HIFU exposure could elevate ROS and free radicals, which might cause chemical damage to tumour cells^62^. In addition, the strong oxidizing activity, chemical inactivity, and biocompatibility of TiO_2_ indicated its potential in cancer treatment^63^.

As mentioned above, chemotherapy-, PDT- and SDT-based cancer cell destruction is mainly based on biochemical processes; however, other physical therapies are also of interest. For instance, ultrasound (US)-triggered TiO_2_-enhanced microbubbles caused mechanical damage to HSC-2 cells^64^, which was revealed to be efficient (the US + TiO_2_ + microbubble group showed the lowest cell survival rate of 14%). This microbubble-mediated mechanical therapy effectively avoids the chemical cytotoxicity of normal cells; nevertheless, this strategy is still in the experimental stage and needs further analysis for future clinical translation.

#### Biomaterial-mediated thermal therapy

Hyperthermia-based treatments also show promise in tumour therapy. Tumour ablation can occur at 42–45°C for 15–60 min or 50 °C for 4–6 min^65^. Hyperthermia can lead to ablation of local tumour tissue after injection of thermal therapy agents into the body and induction of these agents to generate heat under external stimuli such as magnetic fields, ultrasound, and light. Traditional hyperthermic treatment utilizes the magnetic field as an external stimulus, which is the so-called MMT. Since Gilchrist first proposed it in the 1950s^66^, MMT therapy has been extensively developed, and due to modern technology, agents can be surface modified with target elements to enhance their concentration at the tumour site. Christopher J. Legge et al.^67^ fabricated anti-αvβ6-conjugated magnetic iron oxide nanoparticles to treat OSCC. When exposed to alternating magnetic fields, nanoparticles generated heat to create localized hyperthermia (>50 °C), resulting in irreversible cancer cell necrosis. In addition, the introduction of anti-αvβ6 enabled nanoparticles to specifically recognize oral cancer cells. This strategy is relatively efficient and biologically safe and holds potential for future cancer treatment.

**Fig. 3:**
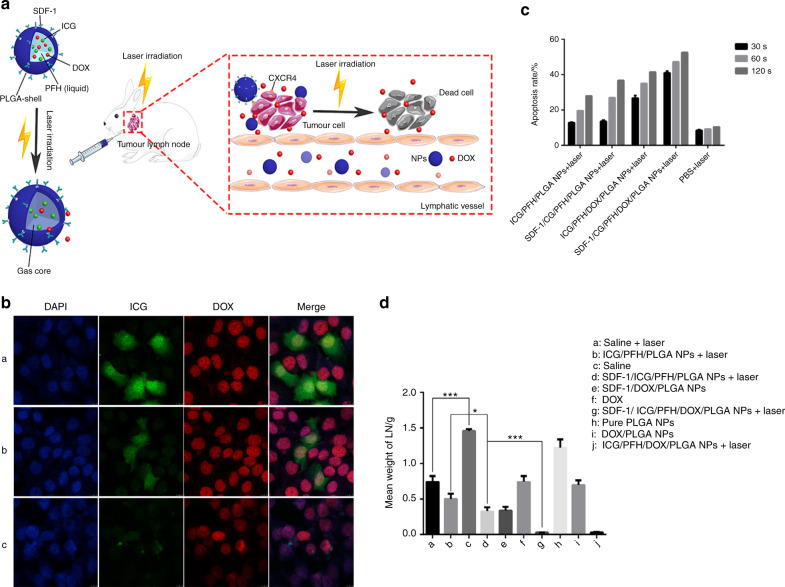
SDF-1-loaded PLGA nanoparticles for tongue squamous cell carcinoma photothermal therapy. **a** The schematic shows the construction of multifunctional PLGA NPs as a visualizable targeted chemotherapy agent for the codelivery of ICG and DOX, lymph node targeting, and chemical-photothermal combination therapy. **b** ICG (green fluorescence) and DOX (red fluorescence) were engulfed by SCC-15 cells, as shown by CLSM (×800 magnification). DAPI stains the nucleus blue. a: Experimental group: SDF-1/ICG/PFH/DOX/PLGA NPs; b: control group I: ICG/PFH/DOX/PLGA NPs; c: control group II: cells were blocked with excess SDF-1 before the addition of SDF-1/ICG/PFH/DOX/PLGA NPs. **c** Apoptosis rates of SCC-15 cells with and without laser irradiation measured by flow cytometry. **d** In vivo therapeutic effect, displayed as tumour weight.^68^ Copyright 2019, Elsevier

Heating malignant tissue appears to be a relatively simple requirement; however, selectively achieving the desired temperature is not a simple technical task. The most important side effect of hyperthermia is the scalding effect on surrounding normal tissues; thus, it is critical to limit the spread of local heat. Conventional hyperthermic methods, represented by MMT, lack sophisticated temperature control equipment or devices. In the case of unsatisfactory biodistribution of therapeutic agents in vivo, a wide range of nonselective heating exposes normal tissues to high-temperature damage. In contrast, using light as an external stimulus can be simple and feasible. Light illumination focused on the diseased site can maximize the retention of local heat and minimize the side effects of hyperthermia. Therefore, PTT is a more promising strategy. However, due to the tunability of light, PTT also shows better application prospects than magnetic field-mediated thermal therapy. Ultraviolet light, visible light, and near-infrared light (NIR) are useful modalities in PTT, among which NIR (650 to 900 nm) is most commonly applied and can effectively penetrate normal surface tissues to deep tumour sites, providing a potential method for affecting tumours growing at sites inaccessible to surgery. ICG serves as a photosensitizer that is responsible for heat generation to induce cell necrosis. Xiong et al.^68^ designed PLGA nanoparticles containing ICG to treat tongue squamous cell carcinoma, and after exposure to a laser (808 nm, 1 W/cm^2^), localized heat was formed due to photothermal conversion. ICG can also be used for photoacoustic imaging, and thus, we could track in real time the location of materials and drugs, simultaneously evaluating the uptake and survival of tumour cells. In addition to ICG, other elements, such as DOX for cancer chemotherapy and SDF-1 for specific targeting, can be used, resulting in combinational cancer therapy. Encouragingly, localized hyperthermia led to accelerated DOX release as well as enhanced tumour cell membrane permeability. Better pharmacokinetics of DOX can be achieved owing to the light-triggered drug release strategy, where drug release is restricted without NIR irradiation but can be triggered rapidly as soon as heat is produced. In addition to photothermal conversion properties, ICG has been reported to produce ROS under irradiation, which can destroy lysosomes to achieve the “lysosomal escape” of the drugs, increase the intracellular concentration of drugs at the tumour site, and further enhance the cell killing effect^69^. With the specific recognition between SDF-1 and CXCR4 (a specific molecule of HNSCC), the anticancer outcome of SDF-1/ICG/DOX PLGA NPs was better than that of other groups, and the systemic toxicity was constrained to a minimum level, indicating a synergetic function of these components (Fig. 3).

## Biomaterials for maxillofacial bone defect repair

**Fig. 4:**
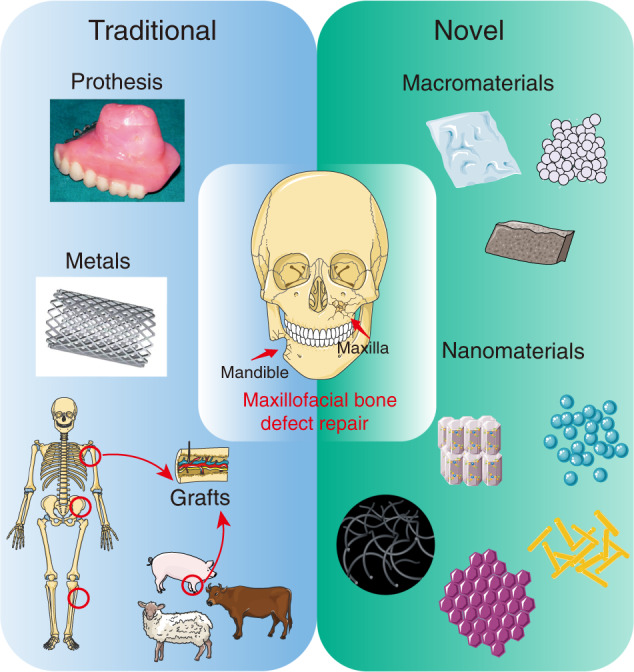
Recent advances in strategies for repairing maxillofacial bone defects after tumour therapy. Traditional techniques include grafts (autologous, allogeneic, xenogeneic), prostheses and metals, while novel strategies are based on bioactive materials, including macroscale (granular, lumpy, flaky material) and nanoscale (nanoparticles, nanocrystals, nanofibres, nanorods) materials. Traditional materials focus on restoring the shape and function of the maxillofacial surface from an “alternative” perspective, while new biomaterials focus on reconstructing bone and surrounding tissues to achieve restoration. The former is currently the main clinical application method, and the latter is in the experimental stage, but this concept is also expected to become a mainstream repair method for patients in the future

Currently, with the increasing demand for maxillofacial aesthetics, bony remodelling has been in the spotlight for its supporting effect in rebuilding other tissues (e.g., skin, muscle, dental implants) in the later stage of functional and morphological reconstruction. Over the last decades, many innovative and effective methods have been applied in the cranial-maxillofacial area. Grafts are clinically admissive approaches for repairing maxillofacial bone defects after treating tumours. However, biomaterials have broad prospects for cranial-maxillofacial bone repair. In addition to traditional bioinert materials (including prostheses and metal materials), bioactive materials are a promising strategy for bone repair owing to their biodegradability, biocompatibility, and bone inductivity. Figure 4 illustrates the advances made in repairing maxillofacial bone tissues, and we will emphasize this issue in the following sections.

### Bioinert materials

Bioinert materials are defined as nondegradable filling materials, which are represented by metals (e.g., titanium, magnesium) and maxillary prostheses (e.g., silicone, polymethyl methacrylate). Maxillary prostheses are well-designed structures determined via 3D reconstruction evaluation that can appropriately fill in the defective bone area. In a case in which a 57-year-old man underwent total rhinectomy and partial maxillectomy^70^, a nasal and obturator prosthesis was needed to restore appearance and function. After recording with a plastic impression compound, a silicone mixture was injected into this model to form the basis of the prosthesis. The connection between the prosthesis and defect area was based on the magnetic effect and water-based adhesion, which indicated that the repair outcome was desirable, with improved mastication, speech, deglutition, and overall appearance. However, silicone-made prostheses still have a risk of infection, collapse and dislocation^71^; therefore, biocompatible materials are preferred and adopted.

Titanium is widely applied in the clinical practice of repairing maxillofacial bone defects owing to its excellent biocompatibility and favourable mechanical properties. Titanium mesh (T-mesh) was applied in mandibular reconstruction after oral cancer treatment due to its suitable strength and stable fixation^72^; in particular, the porous structure of T-mesh supported rapid vascularization, and the bone integration period was shortened. Moreover, T-mesh combined with autologous composites can result in beneficial repair outcomes, such as the rectus abdominis myocutaneous free flap^73^ implanted on T-mesh after its fixation in advance in vivo. One year after the surgery, no recurrence or complications (e.g., infection, T-mesh breakage) were observed, and the maxillofacial appearance was favourably maintained. However, the bonding between bone and bioinert materials is mainly mechanical, and thus, the normal function may be impaired compared with that of the original structure. Stress shielding^74^, resulting from the inconsistent Young’s modulus of bones and implants, would lead to bone atrophy.

Strategies for the modification of titanium are mediated by adding specific elements, such as bioactive molecules (including PRF^75^ and BMP^76^) and inorganic metallic ions (e.g., magnesium^77^). Via modification, the surface morphology of titanium has shown goal-directed reformation, which is more suitable for surrounding cells (such as BMSCs) to adhere and facilitate osteogenic differentiation (enhanced alkaline phosphatase (ALP) expression and calcium deposits). As mentioned above, the regenerative function of platelet-rich fibrin (PRF) was reported to be attractive. Jonas Lorenz et al.^75^ synthesized a novel composite material by attaching PRF to a titanium mesh and utilized it for repairing mandibular resected defects. The bioactivity of titanium mesh was improved due to the incorporation of PRF, which showed us new methods for adjusting titanium’s properties, and PRF-based modification might be beneficial for future bone graft improvement.

To date, although bioinert material is a widely utilized defect-filling tool, its application still conflicts with “restoring the original bone”. Bioinert materials are nondegradable and act as a barrier to the growth of newly established bone. As a result, the defect site cannot be perfectly restored to its normal structure. Fillers or implants also have a risk of complications such as infection, disintegration, and dysfunction. Therefore, introducing more effective and superior bioactive materials has become one of the new targets in the field of maxillofacial bone repair, which will be highlighted in the next section.

### Bioactive materials

Bioactive materials show desirable biocompatibility and biodegradability and can form an organic combination with bone tissue, thus facilitating subsequent bone induction and bone integration. The advantages and disadvantages are compared in Table 1. Bioactive materials can also be divided into two categories: cell-free and cell-containing strategies; herein, we will focus on these two approaches.

**Table 1:** A brief comparison of autologous, bioinert and bioactive materials

Material type	Source	Advantages	Disadvantages	Refs.
Autologous	Fibula; Ilium, etc.	No immune rejection; Quick healing; Mature clinical application	Complications due to bone loss at the donor site	^151–154^
Bioinert	Silicone; Titanium, etc.	Designable; Quick but temporary restoration	Limited-service life; Dislocation; Infection	^70,72,155^
Bioactive	Natural (HAP, CaP, chitosan, etc.); Synthetic (PEG, PLGA, etc.)	Biocompatible; Biodegradable; Designable	Lack of clinical application; Nonstandardized evaluation; Undesirable mechanical properties; Long healing period	^102,156,157^

#### Cell-free strategy

The maxillofacial bone defect repair mechanism via cell-free scaffolds creates a three-dimensional microenvironment^78^ for adherence, proliferation and differentiation of the surrounding cells. In addition, the scaffolds degrade while newly born bone forms. Moreover, bioactive molecules released from scaffolds can stimulate osteogenesis. To accomplish these targets, designed scaffolds should be equipped with specific properties, including excellent biocompatibility and biodegradability, suitable porosity, mechanical properties, desirable angiogenic properties and osteoinductivity. Herein, what follows is the introduction of the cell-free strategy, and we will emphasize some biomaterials with potential.

Generally, biocompatible and biodegradable materials are derived from natural and synthetic resources. Collagen^79,80^, chitosan^81,82^, gelatine^83^, and hyaluronic acid^84^ are representative natural biomaterials with a desirable cranial-maxillofacial bone regenerative capacity because they share a similar ECM with the host and are suitable for cell migration, proliferation and osteogenic differentiation. Interestingly, the in vivo metabolic components of these natural biomaterials are also needed in the bone tissue reconstructive process. Currently, bioceramics and biopolymers are approved by the Food and Drug Administration (FDA) for cranial-maxillofacial utilization. Calcium phosphate (CaP)-based bioceramics^85,86^ have received widespread application, especially injectable CaP, which is shows strong formability and flexibility. Nevertheless, the degradation of injectable CaP is limited, thus hindering the growth of newborn bones, so introducing porous materials with an enhanced degradation rate would be necessary. In addition to natural sources, synthetic polymer-based biomaterials derived from a series of polymerization and crosslinking processes are designed purposefully with expected properties and functions, among which PLGA^87^ and PCL^88^ with nontoxic, gelling, filming, and capsuling properties have received widespread use. The properties of synthetic materials alone are slightly inferior, but when natural materials are introduced, a better bone repair result is realized. A CaP/PLGA scaffold^89^ was designed with a high porosity and low immune rejection from giant cells due to the CaP osteoinductive surface. In addition, this microporous structure composite was applied in two clinical cases and could function well in the implantation area with substantial bone volume and vessel formation, establishing a paradigmatic model for oral-maxillofacial bone repair in the future.

A macroporous structure^90^ (refers to pores over 100 μm) allows angiogenesis and the migration of bone cells, which imitates the bone tissue structure and can result in excellent bone repair outcomes. The quintessential scaffolds share a similar or identical structure and composition with the bone itself. For example, HAP^91^, consisting of approximately 65% bone, has a suitable natural structure for bone regeneration. HAP can also combine with other material components to enhance bone tissue engineering. In a study^92^ comparing the mandible bone defect repair of two materials, three-dimensional hydroxyapatite/poly-D/L-lactide (3D-HAP/PDLLA) and beta-tricalcium phosphate (β-TCP), 3D-HAP/PDLLA had pore sizes ranging from 40 to 480 μm (average pore size = 170 μm), was suitable for bone regeneration and was superior to β-TCP in facilitating osteogenesis and angiogenesis. The porous structure formed by association with a morphologic material, such as microspheres, may also be useful. To overcome the slow degradation rate of CaP, Habraken et al. found that by adding PLGA microspheres to CaP, the porosity was effectively elevated from 41 to 60% (PLGA/CaP, 10:90) and 69% (20:80)^93^, which could accelerate the bone forming process.

**Fig. 5:**
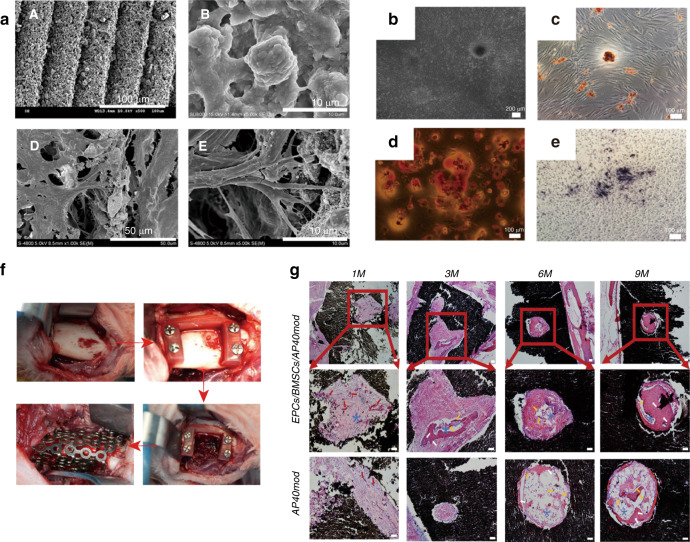
Bioactive glass ceramics by DLP printed containing EPCs/BMSCs for rabbit mandible defects bone repair. **a** Cross-sectional micrographs of **A** P40mod. **B** Microstructure of AP40mod. **D** and **E** SEM images of AP40mod seed cells; **b** BMSCs exhibited fusiform–like morphology. **c** Alizarin red staining. **d** Oil red staining. **e** ALP staining. **f** Surgical procedure. **g** HE results. Red arrow indicates neovascularization, yellow arrow indicates osteoblasts, white arrow indicates bone cells, * indicates bone marrow.^118^ Copyright 2020, Elsevier

Additionally, osteoinductive factors (e.g., BMP-2^94,95^, FGF-2^96^, IGF, PDGF-BB^96,97^) have essential roles in promoting osteogenesis. BMP-2, a member of the TGF-β family, can stimulate DNA synthesis and cell osteogenic differentiation^98^ via the Smad/MAPK pathway. When these factors are combined with other bioactive materials, the synergistic effect would be enhanced. Boda et al.^99^ combined calcium-binding BMP-2 mimicking peptides with PLGA-collagen-gelatine nanofibre segments and then applied this composite in a rat periodontal bone defect repair model, which showed that bone density and volume were elevated ~3 times compared to those of the control group. Interestingly, these nanofibre fragments also served as a “drug container”, enabling 4 weeks of sustained BMP-2 release. However, considering the high cost and side effects of BMP, we found that bone-forming peptide-1 (BFP-1)^100^, a fragment existing in the immature area of BMP-7 protein, was more effective than BMP with enhanced osteogenic inductivity. Herein, Li et al. combined BFP-1 and aspirin-loaded liposomes (Asp@Lipo) with a PCL scaffold for application in a rabbit cranial defect animal model^88^. When cocultured with scaffolds in vitro, human mesenchymal stem cells (hMSCs) showed an increased osteogenic differentiation capacity. In addition, elevated bone formation (45.12% bone volume) was observed with the participation of BFP-1 and aspirin released from the scaffold compared with each single drug (BFP-1, 28.84%; Asp@Lipo, 35.65%), which suggested that the dual drugs acted synergistically on bone regeneration. Moreover, synthetic anabolic bone-forming drug conjugates (C3 and C6), consisting of an enhanced bone activator of the prostaglandin E2 receptor and inactive bisphosphonate, were reported to be beneficial for bone regeneration. In one study, C3 and C6 were encapsulated within monetite (consisting of β-tricalcium phosphate (β-TCP) and monocalcium phosphate monohydrate (MCPM), respectively) to improve osteoinductivity in a mandibular bone defect model in Sprague-Dawley rats^101^, and the results showed that monetite+C6 resulted in the highest bone repair percentage (~42%, compared with ~32% in monetite+C3 and ~22% in the control group) at 4 weeks. This research provided proof-of-concept for a novel therapy that combined bone anabolic drugs with biodegradable monetite, and it also identified a promising therapeutic direction for bone repair after maxillofacial tumour surgery in the future (Fig. 5). Furthermore, concentrated growth factor (CGF)/fibrin composite scaffolds can promote the repair of jaw defects, which was proven by Fang et al.^102^ through a clinical trial: compared with those of the control group (only using Bio-Oss bone powder), the postoperative bone mineral density, serum bone alkaline phosphatase (BAP) and osteocalcin levels of patients with jaw defects were found to be significantly increased in the experimental group (using CGF/fibrin composite associated with Bio-Oss bone powder). CGF^103^ is a platelet concentrate composed of platelets, various growth factors (VEGF and transforming growth factor (TGF-β), etc.), and a large amount of fibrinogen, etc. Among them, growth factors can stimulate the proliferation and differentiation of osteoblasts, and the fibrin structure with good elasticity and large gaps can bind and slowly release growth factors, thereby promoting bone tissue repair and new bone formation^104^. Additionally, CGF fibrin is easy to prepare and has strong biocompatibility, which suggests that CGF fibrin is a promising maxillofacial bone repair material. In addition to osteogenic factors, strategies combining bioactive materials and immunomodulatory agents have been reported. Zheng et al.^105^ implanted decellularized bone matrix (DBM) into cranial bone defects with the delivery of IL-4. As a result, well-regulated macrophage polarization was observed, leading to a pro-healing microenvironment with enhanced osteogenesis and angiogenesis, which suggests that an immunomodulatory strategy shows potential to effectively regulate the process of bone regeneration.

#### Cell-containing strategy

Unlike the cell-free strategy, this technique transplants materials and seed cells together to the defect site for bone repair, which is an important part of tissue engineering. When seed cells (mostly MSCs) are cultured with materials in vitro, the cells can quickly adapt to the in vivo bone microenvironment, and the process of vascularization and osteogenesis is accelerated.

Even if bioinert materials are well designed, it takes a long time for mesenchymal stem cells to migrate to them, adhere to the materials and proliferate, during which time the risk of infection and integration may be increased. However, bioactive materials can be utilized to improve the poor osteogenic performance of bioinert materials. For instance, Scott P. Bruder et al.^106^ seeded isolated bone marrow-derived mesenchymal stem cells (BM-MSCs) into a scaffold structure made of bioactive materials (hydroxyapatite cylinder) and applied the structure to the femur transplantation region, resulting in a superior tissue structure and biomechanics in the BM-MSC-seeded group compared to the control group. Through a control experiment on sheep, Herve Petite et al.^107^ found that the BM-MSC group built a joint interface between the implant and the organism, while the periosteum-covered group or the scaffold (porous ceramic scaffolds)-only group did not show greater mechanical strength. However, the limited sources and difficulty in separating BM-MSCs lead to limitations in recent research and the clinic. Thus, adipose-derived stem cells (ASCs)^108^ are considered to be an alternative solution, although the osteogenic ability of ASCs and the performance comparison to BM-MSCs remain to be studied^109^. In addition, certain oral stem cells, such as human periodontal ligament stem cells/HPLSCs^110,111^, can differentiate into osteoblasts and grow on scaffolds under osteoinductive conditions in vitro. Due to the consistent source of maxillofacial bone, oral stem cells are more likely to differentiate into maxillofacial tissue in vitro. Moreover, there are studies on the use of homology to induce the differentiation of oral stem cells to repair maxillofacial nerves and muscles^111^.

Song et al.^112^ designed an injectable calcium phosphate cement (CPC) scaffold carrying cell-encapsulating alginate-fibrin microfibres (Alg-Fb MF) to repair mandibular rami defects in athymic nude rats, which significantly promoted osteogenesis with an osseous bridge (three times as much as the control group) at 12 weeks, and more neovascularization was observed histologically. Moreover, after in vitro culturing, the results of the Cell Counting Kit-8 (CCK-8) assays and Alizarin Red S staining (ARS) indicated that the CPC-MF scaffold maintained high hBMSC viability and osteogenic differentiation potential, indicating a promising option for cell delivery in future maxillofacial bone defect repair. In addition, as a widely applied biomaterial approved by the FDA for maxillofacial utilization, CPCs containing other stem cells with osteogenic potential were reviewed as promising candidates for dental and craniofacial bone tissue engineering, particularly prevascularization that facilitated vascularization in vivo as soon as the composite was implanted^29^. Interestingly, the other properties of CPC, such as tailored micro- and nanoscales that offer cell adhesion and protein adsorption, are of interest^113^. In addition, as an injectable material^114^, CPC has fine mechanical properties that could bear weight in tissues such as bone and enlarge the bone-material contact area for better structural rebuilding^115,116^.

**Fig. 6:**
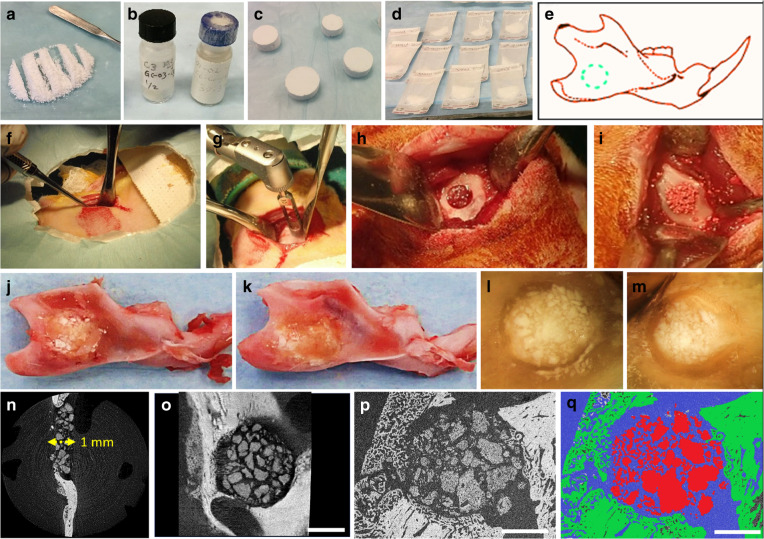
Monetite granules with bone anabolic drug conjugates (C3 and C6) in repairing rat mandibular defects. **a** Mixture of β-tricalcium phosphate (β-TCP) and monocalcium phosphate hydrate (MCPM) powders. **b** Vials of C3- and C6-conjugate solutions. **c** Blocks of Brushite cement grafts prior to crushing and conversion to monetite. **d** Monetite granules (0.25–1 mm) after autoclaving in sterile pouches. **e** Schematic illustration of the rat jaw defect model. **f**–**h** Surgical incision and drilling of critical sized surgical defects (4.3mm diameter). **i** The surgical defect packed with monetite granules. **j**, **k** Mandibles with defects filled with monetite granules retrieved after 2 and 4 weeks of implantation. **l**, **m** The appearance of resin-embedded polished bone blocks for imaging with B-SEM. (n and o) Micro-Ct image. (p and q) B-SEM images.^101^ Copyright 2020, Wiley

In addition to CPC, other materials are of interest for cell culturing in maxillofacial bone repair. A novel alginate-based hydrogel scaffold synthesized by Marie Naudot et al.^117^ was designed for bone marrow mesenchymal stem cell (BMSC) in vitro culture, and then, the composite was engrafted into palate lesions of Sprague-Dawley rats. According to the μCT analysis, the mean lesions in the scaffold + BMSC group were smaller than those in the other groups, showing a fascinating bone healing effect. However, the repair outcome would be compromised due to the underdeveloped mechanical strength of the scaffold; therefore, materials with desirable mechanical properties would be preferred. Xu et al.^118^ designed a bioactive glass ceramic (AP40mod, consisting of SiO_2_, P_2_O_5_, CaF_2_, TiO_2_, etc.) carrying endothelial progenitor cells (EPCs) and BMSCs for rabbit mandible bone defect repair. As shown in Fig. 6d, e, EPCs and BMSCs revealed desirable ductility and attachment when cocultured with AP40mod in vitro, presenting as paving stone-like EPCs attached to long fusiform BMSCs. CCK-8 assays demonstrated the excellent cell biocompatibility of AP40mod, and the relatively higher ALP expression in the 2:1 group shown in Fig. 6L indicated an effective osteogenic induction. In addition, the excellent mechanical properties (with a bending strength of 52.7 MPa) were notable and suitable for supporting bone structure. In the animal experiment, superior bioactive properties and increased osteogenesis and angiogenesis were also histologically observed (Fig. 6).

In clinical practice, cell therapy for inducing maxillofacial bone regeneration has also been verified. Atrophy of hard and soft alveolar tissue results from maxillofacial bone defects, as shown by a reduction in both the horizontal and vertical dimensions or severe mandibular ridge resorption. MSCs are widely applied cells with fundamental sources and the ability to differentiate into various tissues (adipose tissue, muscle, bone, etc.). Eleven patients with severe mandibular ridge resorption (aged 52–79 years) were included in a clinical study^119^. Then, the researchers aspirated BMSCs from the posterior iliac crest, followed by expanding the adherent cells in culture medium with human platelet lysate. BMSCs were seeded on the biphasic calcium phosphate (BCP) granule scaffold in vitro, and then, this composite was placed subperiosteally onto the resorbed alveolar ridge. After 4–6 months, new bone formation was assessed clinically and radiographically, and the safety and feasibility of this method were evaluated. With the participation of the BCP/BMSC composite, accelerated bone formation was observed, and the newly regenerated bone was adequate for dental implant installation. In addition, no adverse events were observed. Additionally, when combined with BCP, MSCs illustrated favourable cell viability, proving that BCP^120^ served as an ideal cell-supportive and bone-inductive scaffold both in vitro and in vivo. The above evidence showed that this method of cocultivation of stem cells and materials in vitro and use in vivo is feasible and is not limited to animal experiments. The therapeutic effect for humans was also notable, providing strong evidence for future clinical trials.

For repairing maxillofacial bone defects, cell therapy is a promising option for addressing the undesirable complications of maxillofacial tumour therapy. Postoperative recurrence remains a challenging issue that might compromise the recovery process. GinPa-MSCs, namely, gingival interdental papilla mesenchymal stromal cells, were reported to be capable of carrying and releasing PTX in vitro^121^, and in an anticancer experiment, the proliferation of human pancreatic adenocarcinoma (CFPAC-1) was inhibited (with an index of 71%) when cocultured with GinPa-MSCs/PTX. This work revealed the new role of MSCs as drug carriers in anticancer applications. Moreover, considering the MSC-based bone regenerative potential, MSC-based technology might yield promising outcomes both in preventing tumour recurrence and bone repair. Nevertheless, stem cell-based tumour therapy has been one of the most controversial topics^122^ in recent years, although there is supportive evidence that MSCs serve as an efficient delivery agent for antitumour treatment (e.g., interferon (IFN), tumour necrosis factor (TNF), and cytosine deaminase). The recurrence of the tumour due to injection of MSCs in cancer patients is also an issue. Furthermore, molecules secreted from MSCs, including VEGF^123^, BMP-2^124^, and IGF-1^125^, have been reported to be responsible for tumour growth, survival and metastasis. Whether the therapeutic outcome of MSCs on tumours is positive or negative remains uncertain; accordingly, this “double-edged sword” stem cell-based therapy requires more proof and data for its clinical translation.

## Biomaterial-mediated antitumour/bone repair strategies

**Fig. 7:**
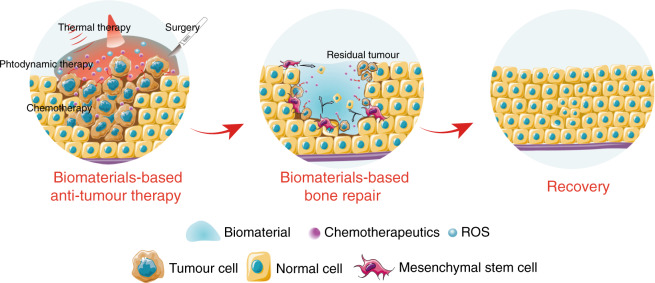
Biomaterial-based tumour therapy and bone repair scheme. Biomaterial-mediated comprehensive tumour treatment involves hyperthermia, chemotherapy, photodynamic therapy, etc., as well as traditional surgical resection. For early treatment, we strive to follow the principle of “no tumour”, and in later repair, the composite material system also plays an important role, including mediating the adhesion, migration, and differentiation of mesenchymal stem cells

The above biomaterials have focused either on maxillofacial tumour therapy or bone repair; nevertheless, the management of tumour therapy and bone reconstruction has not been systemically illustrated. Identification of a treatment strategy that combines tumour therapy with subsequent bone repair can reduce the treatment periods and trauma that patients would undergo, and this combined approach is expected to be an integrated tumour therapy strategy. The basic scheme of biomaterial-based tumour therapy and bone repair is shown in Fig. 7. We elucidated this strategy in detail and analysed its current situation, challenges and prospects in the following section.

Hydroxyapatite (HAP)^126^ is the major component of human and animal bones and is known as an osteoinductive component for stimulating and facilitating bone regeneration^136^. Interestingly, a hydroxyapatite (HAP) nanoparticle-based anticancer drug delivery system^127^ was designed for treating OSCC. Taku Murata et al. tried to encapsulate zoledronic acid (ZA), CDDP and carboplatin into HAP nanoparticles, which were shown to be effective in anti-cell experiments. The reported direct anticancer effect of nHA attracted our interest, and Zhang et al.^128^ proved its feasibility by combining nHA with a titanium structure applied in a tumour-related bone defect repair model. The activation of the immune system as well as mitochondrial-related cell apoptosis revealed the nHA-mediated antitumour mechanisms and accelerated bone formation via nHA facilitation, indicating the dual functional role of nHA and its potential in managing tumour treatment and bone repair. More importantly, how to design this or related systems to function is of interest. Researchers must ensure the tumour treatment outcome in the first stage while maintaining the progress of bone repair in the second stage. Given chemotherapeutics as an example, we can accurately control the local concentration of the drug by studying its slow-release kinetics to ensure that there is a high concentration to inhibit tumours in the early stage. In the later stage, the concentration can be maintained at a low level, which can not only inhibit the residual tumours but also protect the bone tissue reconstruction process.

As we discussed above, strategies for a one-time solution for killing the remaining cancer cells as well as facilitating bone regeneration are more favoured. Unfortunately, direct proof of a maxillofacial tumour-related bone defect repair model has not been reported. Nevertheless, the reference value of the other bone-related antitumour experiments could also inspire us. Many 3D-printed bioceramic materials^129^ with fascinating biological performance have been proven to have strong bone reconstruction potential. In addition to bone regeneration, multifunctional bioceramic materials can simultaneously promote tumour therapy and bone repair. Herein, we can apply these scaffolds to bone defect repair due to tumour therapy. Ma et al.^130^ designed a 3D polydopamine-modified bioceramic (DOPA-BC) scaffold to eliminate residual carcinoma tissue via PTT and simultaneously support the adherence and proliferation of rabbit bone mesenchymal stem cells (rBMSCs). This dual functional scaffold was tested through in vitro examination after NIR laser irradiation and then maintained localized controllable hyperthermia (over 50 °C, for 4–6 min) that was sufficient for cancer cell clearance. In addition, the in vivo animal experiment proved its feasibility, and the DOPA-BC + NIR laser group showed relatively outstanding anticancer effects according to H&E staining and immunohistochemical analysis. We believe that in the future, the success of more models, as well as solid data, will be reported. Similarly, graphene oxide (GO) is a classical photothermal material widely utilized in antitumour therapies; based on that, a GO-modified bioceramic scaffold was fabricated by Wu et al.^131^ Under irradiation, GO effectively converted light energy into heat to create a high-temperature localized area in the tumour, which played a role in tumour ablation. In the later stage of treatment, this bioceramic material can also fill the bone defect left by tumour ablation to enhance the bone repair effect. In addition, in vitro experiments have proven that MSCs can form an organic combination with materials, thus improving the efficiency of bone formation. Therefore, this is also a good model reference for bone repair after maxillofacial tumour treatment.

In addition to these materials, hydrogels with multiple functions (including anti-inflammatory effects^132^, antibacterial effects^133^, tumour treatment^134^, tissue repair^135^, etc.) have long been applied, and injectable hydrogels have to potential to reach the targeted area without surgical harm. Luo et al.^136^ designed a novel injectable hydrogel composite encapsulating CDDP and polydopamine (PDA)-modified nHA for antitumour PTT and bone reconstruction. Sustained release of CDDP was observed via abundant groups of PDA. Additionally, PDA served as a PTT agent for localized hyperthermic generation to ablate tumours. Furthermore, enhanced attachment, proliferation and osteogenic differentiation of BMSCs in vitro were observed, further proving the bone regenerative induction capacity of this hydrogel composite. Herein, this research has strongly proven the promise of multifunctional hydrogel-based materials in antitumour and bone repair, which also strengthened the effectiveness of this hybrid therapy platform. In addition, soft materials (such as hydrogels^137^) have desirable adhesion for local administration in a relatively humid environment (such as the oral cavity) and can effectively combine with local tissues and continuously improve the efficacy of tumour treatment.

In clinical practice, when we treat patients with maxillofacial malignant tumours, the tumour type, stage, degree of compression or invasion of surrounding tissues and organs, and the patient’s demands are necessary premises to consider before formulating a personalized, comprehensive treatment plan. In general, “surgery + radiotherapy/chemotherapy” is a widely adopted plan; for additional management, strategies were described in Section 2.1. Likewise, when we discuss biomaterial-mediated treatment options, we also need to consider the specific conditions of tumour patients to better carry out clinical translation.

In addition, in fundamental experiments, most maxillofacial tumour researchers adopt direct treatment in a subcutaneous tumour model, while few maxillofacial tumour cases adopting surgery + postoperative radiotherapy + chemotherapy in the clinic have been reported. However, some bone-related tumours have certain reference values, such as osteosarcoma, which occurs often in the knee joint area, but there are also reported cases in the maxillofacial bone. Therefore, Yu et al.^138^ designed sunitinib and Ce6 via redox-responsive zwitterionic hydrogels to prevent the recurrence of osteosarcoma after surgical resection and showed that those in the treatment group survived, while those in the other groups almost died approximately 60 days after surgery owing to tumour recurrence. Therefore, single surgical treatment might not be sufficient, and surgical resection + biomaterial local treatment (chemotherapy, immunotherapy, etc.) is used in the clinic and might be efficient in inhibiting postoperative tumour recurrence and prolonging the lifespan of mice. Our team is now working on the establishment of bone tumour models in situ, PTT and chemotherapy treatment, and evaluation of bone tissue repair at later stages in an attempt to design a biomaterial-based platform managing bone-related malignant tumours through the entire therapeutic process.

Surgical resection + radiation/chemotherapy is a common clinically adopted treatment scheme, and for biomaterial-mediated therapies, surgery is necessary to eliminate carcinoma tissue to provide space for new bone formation and integration. After tumour surgery, biomaterials such as alloys, titanium, and bioceramics are applied as maxillofacial bone substitutes, and adjuvant treatments such as systemic radiotherapy and chemotherapy are used to inhibit recurrence. However, the prognosis and bone repair are sometimes unsatisfactory owing to the poorly controlled doses of radiotherapy and chemotherapy. Herein, localizing postoperative radio/chemotherapy could effectively guarantee repair outcomes, which would be realized via biomaterial-based platforms (e.g., sustained release of chemotherapeutic drugs, control of systemic toxicity, centralization, and high efficacy of treatment). As explained above, when the two functions of inhibiting tumour and bone regeneration are integrated into one biomaterial platform, the degradation rate of the material, the control of drug concentration, and the regulation of local immune-inflammatory response are all factors that need to be considered. At present, few studies of this type of treatment are available, but we still hope the concept we put forward here could promote future research.

## Perspectives and Challenges

With the rapid development of advanced biomaterials, the strong potential for maxillofacial tumour therapy and subsequent bone tissue repair has been demonstrated. However, various problems remained unsolved.

First, compared with those of traditional autogenous bone flap transplantation the mechanical strength, modulus, and degradation rate of biomaterials needed for dynamic changes^139^ in bone reconstruction in terms of the different situations of the patient (immune diseases, blood diseases, etc.) are unclear. As mentioned before, a strategy combining both cancer therapy and bone tissue regeneration has been put forward^54^; although it is theoretically promising, there is no direct evidence of in vivo experiments at present, and changes in the body (pH, temperature, enzyme, cell, etc.) can be difficult precisely predict. Herein, before implantation, accurate design of the proportion and material structure is essential. For instance, tumour-specific recognition molecules^140,141^ or environmental change-sensitive mechanisms^142^ can be added to the system so that materials can react accordingly after contacting the local tumour microenvironment, control the release of chemotherapy drugs, and achieve the effect of killing the remaining tumour cells and reducing systemic toxicity.

There is one more point: when the maxillofacial bone defect area is extremely large, some biodegradable materials with limited strength and mechanical properties may not be enough to support the surrounding tissue; therefore, combining biodegradable materials and nonbiodegradable materials temporarily would be a good option^143^; however, nonbiodegradable material does not meet the requirements of “restoring the original bone”, and extra trauma may inevitably occur along with secondary surgery. Bioadhesives^144^ seem an advisable choice that can temporarily stabilize implant materials and allow time for bone integration. Ideally, we need novel materials with structures and properties closer to bone tissue, thereby meeting the needs of support at the early stage of repair, that can harmlessly degrade in vivo with metabolites that are capable of being used as raw materials for bone tissue reconstruction.

Finally, the outcome of combining anticancer agents and bone regenerative elements in one system is unclear, owing to the possible contradictory solubility^145,146^, charge effect^147^, chemical interactions^148^, etc. The in vivo release kinetics are more unpredictable when considering two more elements. For instance, DOX is hydrophilic, while curcumin is hydrophobic; accordingly, Karavasili et al.^55^ designed a self-assembling peptide hydrogel for localized drug codelivery, and both the in vitro and in vivo drug release evaluations illustrated that DOX and curcumin release corresponded to their aqueous solubilities (DOX release almost converged in 24 h, while a relatively prolonged curcumin release was witnessed in 19 days). In addition, in vivo model construction is difficult due to the complicated structures^149^ in situ, and in fact, the maxillofacial bone structure differs from cranial structures, which many studies chose as the model; therefore, the material implantation plan needs to be carefully considered, avoiding important nerves and blood vessels. Indispensably, reconstruction of mandibular defects should provide enough room for later dental implants. Moreover, the success of tumour treatment and bone tissue reconstruction is related to the in vivo performance of the biomaterial platform system. For instance, whether the drug release concentration and degradation cycle of the material platform are adequate defines the tumour growth inhibitory efficacy during the tumour treatment period. In addition, biomaterial-triggered local immunity^150^ is effective for tumour treatment, but in the repair phase, the immune-inflammatory response should be properly controlled to ensure the repair effect; thus, it is critical to determine the balance of the material’s in vivo performance during the entire treatment process.

To date, the prospects of biomaterials are promising, and future works need to address the following:

1. Develop and design more biomaterials with superior performance;
2. Reveal and summarize commonalities of biomaterials between oral cancer therapy and maxillofacial bone repair;
3. Obtain multifunctional materials for various applications, including tumour management, stimulation of bone regeneration, and antibacterial and other properties;
4. Build more in vivo models, thus obtaining sufficient evidence to promote oral maxillofacial bone reconstruction after tumour treatment.

## Conclusion

In this review, we have suggested a novel biomaterial strategy, in theory, that takes both maxillofacial tumour treatment and bone regeneration into consideration. The functional components of each part were introduced in detail, and then, the advantages and disadvantages of each part were analysed. In the future, superior biomaterials with the functions of both antitumour and osteogenic repair will substantially decrease the hospitalization time of patients, thus reducing the possibility of infection during hospitalization and ultimately improving the prognosis of patients, although more work is needed from materials scientists, oncologists, surgeons and other researchers.
